# Novel Wireless Sensor System for Monitoring Oxygen, Temperature and Respiration Rate of Horticultural Crops Post Harvest

**DOI:** 10.3390/s110908456

**Published:** 2011-08-30

**Authors:** Mette Marie Løkke, Helene Fast Seefeldt, Gareth Edwards, Ole Green

**Affiliations:** 1 Department of Food Science, Aarhus University, Aarslev DK-5792, Denmark; E-Mails: mettem.loekke@agrsci.dk (M.M.L.); helenef.seefeldt@agrsci.dk (H.F.S.); 2 Department of Engineering, Aarhus University, Tjele DK-8830, Denmark; E-Mail: gareth.edwards@agrsci.dk (G.E.)

**Keywords:** wireless sensor networks, vegetable, post harvest research, respiration rate, temperature

## Abstract

In order to design optimal packages, it is of pivotal importance to determine the rate at which harvested fresh fruits and vegetables consume oxygen. The respiration rate of oxygen (RRO_2_) is determined by measuring the consumed oxygen per hour per kg plant material, and the rate is highly influenced by temperature and gas composition. Traditionally, RRO_2_ has been determined at discrete time intervals. In this study, wireless sensor networks (WSNs) were used to determine RRO_2_ continuously in plant material (fresh cut broccoli florets) at 5 °C, 10 °C and 20 °C and at modified gas compositions (decreasing oxygen and increasing carbon dioxide levels). Furthermore, the WSN enabled concomitant determination of oxygen and temperature in the very close vicinity of the plant material. This information proved a very close relationship between changes in temperature and respiration rate. The applied WSNs were unable to determine oxygen levels lower than 5% and carbon dioxide was not determined. Despite these drawbacks in relation to respiration analysis, the WSNs offer a new possibility to do continuous measurement of RRO_2_ in post harvest research, thereby investigating the close relation between temperature and RRO_2_. The conclusions are that WSNs have the potential to be used as a monitor of RRO_2_ of plant material after harvest, during storage and packaging, thereby leading to optimized consumer products.

## 1. Introduction

Postharvest research and technology refer to the handling, sorting, storage, transportation and sale of plant material from harvest to consumption. Special focus is on quality changes and loss reduction in the postharvest chain of fresh fruits and vegetables. Although harvested, the fruits and vegetables are still alive; they respire, consume oxygen and emit carbon dioxide. The respiration rate is the rate at which oxygen is consumed or carbon dioxide is generated. The rate is defined either as the consumed mL of oxygen (RRO_2_) per kg per hour or as the generated mL carbon dioxide (RRCO_2_) per kg per hour and is a key measure in post harvest research [1]. Respiratory parameters are correlated to the rate of deterioration of the plant material, and these parameters are especially important when designing modified atmosphere packaging (MAP), where the permeability of the packaging material must be designed to match the respiratory parameters [2–5]. Fresh fruit and vegetable respiration is affected by temperature, wounding, gas composition, and physiological factors such as pathogen attack and varietal differences [6–9]. Respiration is measured in either closed/static systems [1,5,10], where the product is kept in closed containers or in flow-through/open systems [10–12] where the gas composite is changed with specified gas concentrations [1] or left at atmospheric conditions. The changes in concentration of O_2_ or CO_2_ are measured over a period of time in the static system as well as in the open system. In the flushed system, the changes are measured between the inlet and the outlet. The initial gas composition can be either atmospheric [13] or composed in a specific manner [5,10,12].

In the literature, the most common method to determine oxygen and carbon dioxide in respiration analysis is gas chromatography [6,7,9,12–14] or electrochemical/infrared gas analyzers [5,10,11,15]. All the methods rely on the removal of a small amount of gas, and they are not continuous measurements, thus resulting in discrete data points. As the development of respiration rate is a continuous process and seldom linear, the discrete measurement points should be chosen with great care [1]. Oxygen partial pressure and temperature have an important impact on fruit and vegetable respiration [1]. Therefore, plant material is often tested under various temperature regimes in controlled climate chambers [5,7,10,12]. However, the exact temperature in the intimate proximity of the plant material has not been determined continuously during these experiments.

Respiration rate measurements would gain from continuously analyzing the gas composition without disturbing the system. Oxygen probes could be an option enabling non-invasive determination of O_2_ in closed systems. In the recent decades, development of Wireless Sensor Networks (WSN) has taken place. In contrast to wired sensors, the obstacle has been to develop hardware that is capable of transmitting data under difficult circumstances, and developing low-cost, long-term energy sources for the sensor nodes [16]. The wireless sensors enable monitoring of processes non-invasively and where cabling is not possible [17]. WSN are in intimate connection with the immediate physical environment allowing each sensor to provide detailed information on environment of material that is otherwise difficult to obtain by means of traditional, wired instrumentation [16]. WSN have been used for different aspects of agricultural measuring, monitoring and control [18], such as precision irrigation, environmental field data collection systems, automated fertilizer applicators, and animal behavior monitoring [19–22].

Recently, WSN have been used within agricultural post harvest research, e.g., storage monitoring [23], or measuring and modeling of processed agricultural biomass quality in storage [24]. Within horticulture, WSN have mainly been used for monitoring environmental and growing conditions in the field or greenhouse. During transport and storage postharvest, WSN are widely used for temperature and psychrometric logging [17]. However, within horticultural post harvest there is still a lack of research and development of WSN [17] for quality monitoring. Only one study determining O_2_ continuously postharvest by using an O_2_ electrode to determine oxygen consumption in packaged tomatoes [25] has been found. Sensors with interesting features for post harvest research are gas/volatile sensors detecting oxygen, carbon dioxide, ethanol or volatile organic compounds, which are emitted from the plant material, reflecting the quality status of the produce. Other interesting features include relative humidity, shock and light impacts [16] and also biosensors used for microbial detection under development [17]. As a novel approach, WSN could be applied to measure respiratory parameters in post harvest technology, such as oxygen consumption. Furthermore, the respiration parameters can be related to the exact temperature experienced by the plant material. Using such WSN enables the continuous measurements of respiration in time-dependent experiments with fluctuating temperature. Finally, the wireless systems make it possible to do measurements without disturbing the system, and thereby preventing introduced changes in gas compositions.

The objectives of this study are: (1) to test novel wireless sensors capable of measuring the temperature and oxygen changes continuously inside 1 L glass jars containing vegetables (broccoli florets) under traditional respiration analysis conditions; (2) to test the communication reliability of the sensors from within climate chambers in changing temperature and oxygen regimes; and (3) to compare the measurements with a standard respiration measurement.

## 2. Experimental Section

### 2.1. Wireless Sensors

The wireless sensors used in this experiment monitor temperature and oxygen concentration. The sensor measures the temperature and oxygen level at specific time intervals and transmits the data wirelessly to a receiver station. The sampling time interval was set to roughly every 1 min. To obtain a long effective transmission communication range with high penetration capability, 433 MHz was selected as the communication frequency for this application.

The sensor is powered by a 3.6 V lithium battery. The oxygen sensor is an O_2_ A3 (Alphasense, Great Notley, Essex, UK) of the galvanic type. The temperature sensor is a TMP36 (Analog Devices, Norwood, MA, USA). The sensor unit consists of a microcontroller, radio, A/D converter, antenna circuit, power unit (battery), temperature sensor, and relative humidity sensor. The nRF9E5 is a single-chip system with fully integrated RF transceiver, 8051-compatible microcontroller and a four-input, 10-bit, 80 kilo samples per seconds (ksps) AD converter. The circuit has embedded voltage regulators, which provide maximum noise immunity and allow operation on a single 1.9–3.6 V supply. The transceiver of the system automatically handles preamble, address, and cyclic redundancy check (CRC). The RF transceiver is accessed through an internal parallel port or an internal serial programmable interface (SPI). The data-ready, carrier-detect, and address-match signals can be programmed as interrupts to the microcontroller or polled via a general purpose input-output (GPIO) port. The nRF9E5 has a radio transceiver for the 433 MHz ISM bands with Gaussian frequency shift keying (GFSK) modulation at a data rate of 100 kbps. The transceiver consists of a fully integrated frequency synthesizer, a power amplifier, a modulator, and a receiver unit. For power saving, the transceiver can be turned on and off under software control. An important aspect of the nRF9E5 node is its ability to set low-level hardware functionality to achieve low-power sleep states.

In this project, each sensor node acted as a transmit-only device in a single-hop broadcast network, and the data was received by a gateway node. To enhance communication reliability, each sensor node actively participated in handshaking communication.

**Figure 1.**
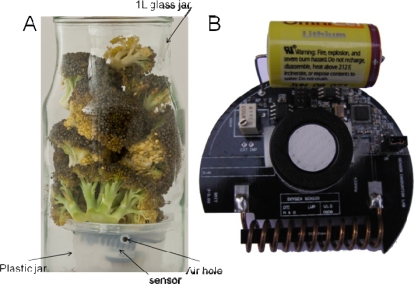
The experimental setup with the O_2_ sensor protected by a plastic housing (**A**). The plastic housing was placed in a glass jar of 1 L, and plant material was placed above and around the housing in the glass jar. Close-up of the sensor (**B**). The white circle in the middle is the oxygen sensor.

The sensors were placed in 6 cm plastic jars (Figure 1) to prevent damage to the sensor from physical (e.g., pressure forces) and chemical stresses (e.g., acidification) during the storage periods. Eight ventilation holes were made just below the lid in the plastic jars. The plastic jar was placed inside a 1 L glass jar, and plant material was placed above and partially around the sensor allowing free movement of gasses. The logged temperature is the temperature inside the glass jar in close proximity to the plant material. Of the 36 sensor nodes tested, eight sensor nodes proved not to transmit signals and one showed obvious false values (O_2_ > 21%). These sensors have most likely suffered from some intruding exudates or condensed water during handling of the glass jars. As the sensors sometimes exert fall-outs during transmission, and some jars were fou und to be leaking gasses, extra replicates should be made in order to ensure enough data when using this kind of sensor nodes in respiration analysis. Only data from an experiment with six of the 36 sensors are reported here, the rest will be reported elsewhere with a focus on the postharvest use.

### 2.2. Respiration Rate Measurements

During postharvest experiments with broccoli in the summer 2010, it was possible to test the usability of the sensors in a real plant produce experimental set-up designed to test the plant respiration rate at three different storage temperatures at either 5 °C, 10 °C or 20 °C in climate chambers (400 L, −9–99øC Binder KB400, Binder, Tuttlingen, Germany). A 1 L glass jar was filled with plant material of either 160 g broccoli florets. The glass jars were closed with a metal lid using a momentum key with a G-force of 5 kg. The lid of the glass jar was equipped with fittings for gas analysis. In all cases a closed system was used, meaning that once closed the glass jars were not opened until all oxygen was consumed by the plant material. The broccoli florets were of the commercial variety ‘Ironman’, and five replicates (glass jars) at each temperature were prepared. Two of the glass jars contained, besides the plant material, also the sensor in the plastic jar, and data from these six sensors (two jars at 5 °C, two at 10 °C and two at 20 °C) are reported here to test the usability of the sensors in a real set-up. Three glass jars were without the sensors but were used for discrete measurements of oxygen and CO_2_ using an electrochemical O_2_ analyzer and infrared CO_2_ analyzer (Checkmate 9900, PBI Dansensor, Ringsted, Denmark). The glass jars were removed from the climate chambers for conventional, discrete respiration measurements using the PBI Dansensor at nine time intervals for the glasses at 5 °C and 10 °C, and at six time intervals for the jars at 20 °C. The time intervals were chosen in order to cover closely the initial respiration period, but also to cover the longer storage period a 5 °C and 10 °C. At all the discrete measurement points, the two sensor-replicates were also removed from the climate chambers in order to handle the five replicates in the same way. Due to the handling of the glass jars, the temperature within the glass jar changed. Furthermore, it is expected that the oxygen levels in the glass jars were equally distributed within the glass jar due to the handling. The jars were measured at room temperature in the laboratory and were outside the climate chamber for maximum 15 min for each measurement before it was returned to the desired temperature and left until next analysis. Discrete measurements are reported in averages of the three replicates.

### 2.3. O_2_ Calibration of the PBI Dansensor

Oxygen measured by the PBI Dansensor was calibrated against gas mixtures of N_2_, 1% O_2_/2% CO_2_ in nitrogen, 10% O_2_/10% CO_2_ in nitrogen, 15% O_2_/25% CO_2_ in nitrogen, and compressed atmospheric air (all AGA Gas AB, Sundbyberg, Sweden). The calibration of the PBI Dansensor had a root mean square error of calibration (RMSE) of 0.12% oxygen.

### 2.4. O_2_ Calibration of the Sensors

The oxygen calibration was made by combining two different data sets: *oxygen calibration1* and *oxygen calibration2*. To obtain controlled calibration points (*oxygen calibration1*); all 36 sensors were placed in one 3 L jar closed with a lid with flushing fittings. The jar was filled with a gas mixture of 10% O_2_/10% CO_2_ in nitrogen (AGA Gas AB, Sundbyberg, Sweden) and placed at 5 °C for 2 h followed by 10 °C for 18 h and 20 °C for 2 h. Then the jar was filled with a gas mixture of 15% O_2_/25% CO_2_ in nitrogen (AGA Gas AB, Sundbyberg, Sweden) and placed at 20 °C for 2 h followed by 10 °C for 2 h and 5 °C for 18 h. The sensors were also tested in a gas mixture of 1% O_2_/2% CO_2_ in nitrogen (AGA Gas AB, Sundbyberg, Sweden), but these tests could not be used for calibration due to the sensors incapability of measuring below 3–4% O_2_. *Oxygen calibration1* therefore consists of two oxygen levels at three different temperatures for each sensor, which alone would result in a two-point oxygen calibration with correction for temperature.

In order to improve *oxygen calibration1* with successive points of oxygen levels, sensor data from other similar post harvest experiments than the one reported in Section 2.2 was used for *Oxygen calibration2*. Two sensors from each of the temperature tested (5 °C, 10 °C and 20 °C) were chosen, and only sensor data points coincident with reference measurement from the same jar were used leading to six data points for each sensor. The two data sets, *oxygen calibration1* and *oxygen calibration2*, were then joint for calibration and resulting in a total 12 data points for each sensor. Each sensor node was oxygen calibrated individually and corrected for temperature in the calibration. Each of the six sensors was calibrated with an R^2^ of 0.97–0.98, and the mean correction for the oxygen signal was 0.76% O_2_. The RMSE of the oxygen calibration was in average 0.77% O_2_ for the six sensors, and this number can be compared with the RMSE of 0.12% from the calibration of the PBI Dansensor oxygen analyzer. The uncertainty of the PBI oxygen analyzer is also a part of the uncertainty of the sensors.

### 2.5. Temperature Calibration of the Sensors

The data for calibration of temperature was made concomitantly with *oxygen calibration1*. For the 3 L jar to be temperature equilibrated, it took approximately four hours. Therefore, for calibration of the temperature, only steady periods of more than four hours were used, leading to four time points—two time-points at 20 °C, one at 10 °C and one at 5 °C for calibration. Means of sensor signal over 1 h were used. The mean correction for temperature was −0.07 varying from −0.14 to 0.005. As the O_2_ signal was found to be influenced by temperature shifts, both the O_2_ signal and the calibrated temperature were used in the O_2_ calibration.

### 2.6. Data Analysis and Determination of Respiration Rate

All calibrations and data analysis were made in MATLAB (MathWorks, Natick, MA, USA) using in-house written scripts. The O_2_ and temperature signals were read into Matlab for each logger in means for every 5 min data-logging. All zeroes were replaced with missing number (NaN) and mis-functional loggers were expelled. Missing data in short intervals during the logging for respiration rate measurements were estimated using linear interpolation. Sensors showing longer intervals of missing data were expelled. The reference data from the gas calibration (*oxygen calibration1*) and the PBI-Dansensor (*oxygen calibration2*) were joined with sensor data and linear regression analysis was used for the oxygen calibration. Estimation of the respiration rate (the oxygen consumption per kg per hour) was determined by calculating the first derivative using a local second order polynomial Satvitzky-Golay smoothing filter with a window size of 17 points corresponding to 85 min. The respiration rate is calculated per weight material per hour.

## 3. Results and Discussion

### 3.1. Stability and Usability of Sensors in Closed Respiration Systems

During respiration analysis in closed systems, the material often starts to deteriorate because the material is still alive and respiring. As anaerobic conditions develop, leakage of moisture and exudates is inevitable. Therefore, the sensor nodes were protected from the material and exudates by placing them in plastic jars with a lid underneath the plant material (Figure 1). The sensors used in this experiment had dimensions that suited the experimental set-up very well, and the sensor set-up did not harm the plant material by pressure or other means. The closed jars were placed in climate chambers, but the extra coverage from the climate chambers did not generally disturb the signals from the sensor nodes to the receiver unit. The receiver was placed up to 3 m from the furthest climate chamber. During the measurements, some sensors had periodically fall-outs, where no data was received, probably due to lack of connection between the sensor and the receiver. The oxygen sensors were found to be well suited for postharvest experiments with regards to the physical set-up used.

### 3.2. Temperature and O_2_ Calibration of the Sensors

**Figure 2.**
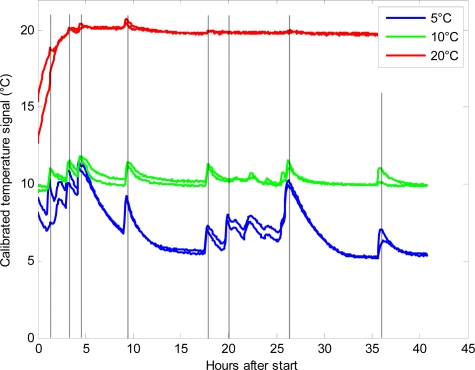
The calibrated temperature signals from six sensors placed at 5 °C (blue lines), 10 °C (green lines) and 20 °C (red lines) at hours after initiating the experiment. The grey, vertical lines represent the time-points were reference measurements were performed.

The sensor nodes transmitted an uncalibrated signal to the receiver. Before exploring the data, the nodes had to be individually calibrated for both temperature and oxygen. The calibrated temperature data are shown in Figure 2. The plot is dominated by sharp peaks, especially visible at 5 °C and 10 °C, and these peaks are ascribed to the opening of the climate chamber doors and the removal of the glass jars for reference measurements. The discrete measurements were performed in an uncooled laboratory at 22–24 °C and that influenced the temperature within the glass jars. Figure 2 also reveals that the equilibration of the temperature of the plant material before the start of the experiment at 20 °C was not sufficient. It is seen that the sensors enable a very fine-tuned and continuous determination of the temperature profile in the glass jar system. This is a major advantage in using the sensors, as the temperature plays a pivotal role in metabolic systems [1].

**Figure 3.**
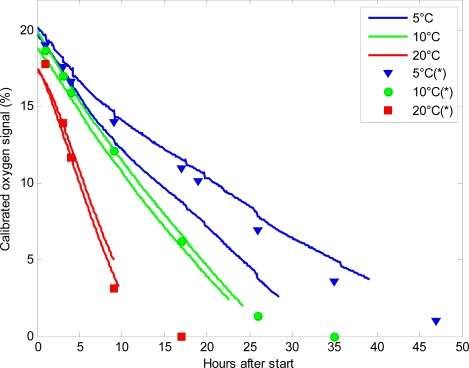
The calibrated oxygen signals from six sensors placed at 5 °C (blue lines), 10 °C (green lines) and 20 °C (red lines) at hours after initiating the experiment, and the corresponding discrete reference measurements made by PBI Dansensor are shown as marks.

Figure 3 shows a plot of the calibrated oxygen signal for six of the sensors placed at 5 °C, 10 °C and 20 °C respectively. When the glass jars were filled with plant material, the lid was immediately closed, and only at the very closing time atmospheric gas composition was present in the jars. The glass jars were filled in another lab with no connection to the receiver from the sensor nodes, resulting in a time lag of approximately one hour from initiating the experiment until signals from the sensors could be received. The sensors placed at 5 °C had almost atmospheric levels of oxygen (20.3%) when the sensors started to log oxygen level, whereas at 10 °C and 20 °C the initial concentration of oxygen was 19.5% and 17.6%, respectively at initiation of logging. These differences in oxygen level after one hour were caused by the very high respiration rate of the plant material at elevated temperatures. This led to consumed oxygen at very high speeds, so even a short lag-period between closing of the lid and logging of the oxygen level would lead to a marked reduction in oxygen. In order to prevent these differences in oxygen levels, it is important to use an amount of plant material that leads to moderate changes in oxygen levels and does not respire too fast. However, the very fast consumption of oxygen found at elevated temperatures further underpins the efficiency and usability of WSN in respiration analysis as it will be possible to log the initial, very fast changes in oxygen level to determine the initial respiration rate. The glass jars were stopped when the plant material was unsuitable for human consumption such as the broccoli seen in Figure 1.

At low oxygen levels, the sensors returned their individual calibrated minimum value. The sensors are, as they are designed now, generally unable to measure oxygen levels below 5%, although some of them could measure O_2_ levels of 2.3%. The mean minimum O_2_ value of the 25 sensors was 4.3% and the highest minimum was 6.9% O_2._ The inability to detect low oxygen levels is a major disadvantage for using the sensors in respiration analysis, as it is often of interest to define exactly when the oxygen drops below 1–2%. This level is, in many cases, the threshold between preservation of the fresh plant material, and anaerobic respiration leading to decomposition of the plant material [6,26]. The used O_2_-chip was the best commercially available chip with low energy requirements suitable for battery operation. It is possible that a chip capable of measuring low oxygen levels with low energy requirements will be available in the future.

### 3.3. Respiration Rates Determined by Sensors

**Figure 4.**
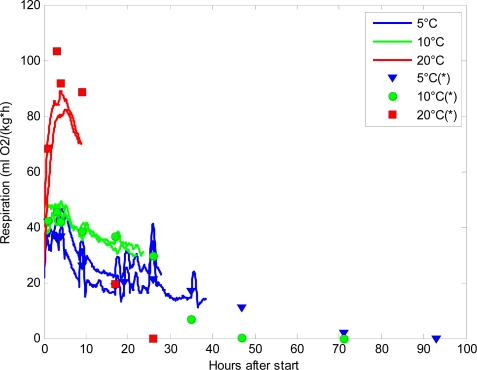
The estimated respiration rates (O_2_) for broccoli placed at 5 °C (blue lines), 10 °C (green lines) and 20 °C (red lines) at hours after initiating the experiment, and the corresponding discrete reference measurements made by PBI Dansensor are shown as marks.

It is of keen interest in postharvest research to follow even subtle changes in respiration rate in order to identify the underlying reasons. Figure 4 shows the estimated respiration rates (RRO_2_) calculated from the continuous measurements of oxygen by the sensors in a postharvest set-up. The sensor measurements are compared to the respiration rates calculated from the discrete measurements made by PBI Dansensor. The respiration rates obtained from the continuous measuring sensors showed fluctuations that are not detectable when using PBI Dansensor (Figure 4). Especially at 5 °C, but also at 10 °C, fluctuations in the sensor respiration rates were seen. At 20 °C, the oxygen level reached levels below 5% oxygen too fast, and therefore it was impossible to get a comparable picture of the respiration reaching a steady state as seen at 5 °C and 10 °C. The fluctuations seen in Figure 4 from the sensors hold information that would never be revealed when using conventional, discrete oxygen measurements.

At 20 °C the discrete respiration measurements were much higher than those found for the sensors. The reason for this might be that the respiration rate is very high at elevated temperatures and irregularities between jars are enlarged. It is known that for high respiring plant material the closed system is difficult to use [1] no matter the method for measuring the oxygen content.

A drawback of the sensors is that they cannot measure carbon dioxide concomitantly with temperature and oxygen. Carbon dioxide is emitted from the plant material during respiration, and especially when respiration changes from aerobic to anaerobic conditions a large increase in carbon dioxide is found. The ratio RRCO_2_/RRO_2_ (the respiratory quotient, RQ) is used to determine the main metabolic substrates oxidized during respiration [6,12] and to determine the point at which anaerobic conditions appear. CO_2_ sensors are available, but commercial available chips with low energy requirements for battery operation have not been found.

### 3.4. The Temperature Dependency of Respiration Rates

**Figure 5.**
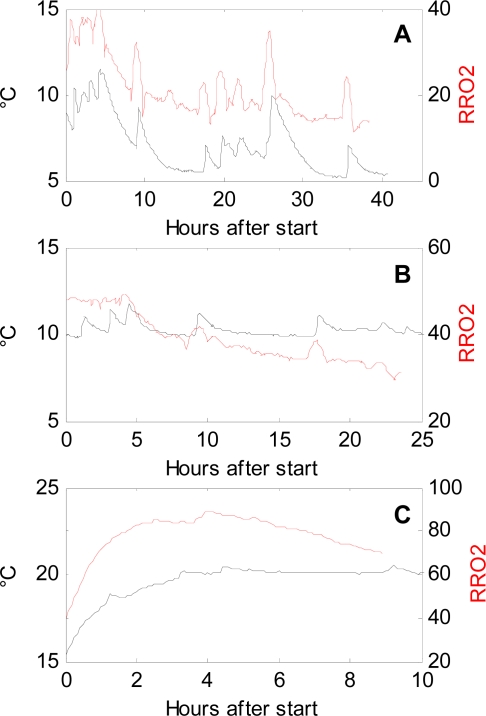
The temperature and respiration rate relationship for each of the three temperatures 5 °C (**A**), 10 °C (**B**) and 20 °C (**C**). In each subfigure the red line refers to the respiration data, whereas the black line represents the temperature data from the same sensor. The respiration rates are only shown until the sensors reached the minimum oxygen level.

The close relationship between temperature and respiration is shown in Figure 5 and has not previously been shown with this high time resolution. Information about temperature and respiration rate are important parameters in software designed to optimize modified atmosphere packaging of fresh fruits and vegetables [4]. Especially at 5 °C where the fluctuations are largest, the relationship is very predominant. It was expected that the respiration rate would increase as a consequence of increasing temperatures. However, Figure 5 shows that the increase in respiration rate was almost synchronic with the increase in temperature. Respiration rates in fresh plant material are very temperature dependent [1], and in this experiment the respiration rate was shown to increase 2–3 times for each 10 °C increase in temperature. Respiration rate curves, based on discrete measurements, determined at temperatures above 10 °C are therefore often too coarse-grained. A temperature increase of 2–3 °C resulted in an increase in respiration rate (RRO_2_) of 10 mL/kg/h. Whether the reason for the discrepancy in the timing is due to physical factors related to the sensors, or to the experimental setup is not clear. The oxygen RMSE of the sensors was much higher than the RMSE of the reference method. By smoothing the calculation of respiration rate, the effects of momentary small irregular changes in the sensor signal were reduced. Considering the match between sensor data and discrete data, despite the high biological variation in the different jars in Figure 3, and looking at the fine-tuned correlation between temperature and respiration rate in Figure 5, the fluctuations in respiration rate following a temperature increase was considered to be meaningful. The calibration of the sensors would be improved by more calibration points; a test with 30 points reduced the uncertainty to approximately 0.5. Aging of the sensors due to the harsh environment with intruding exudates and condensed water in postharvest experiments and biomass monitoring might also disturb the calibration. The effect of the harsh environment on aging is considered to be substantial compared to the aging due to high levels of CO_2_ and humidity. In these kinds of experiments, the lifetime of the sensors must therefore be considered to be lower than in experiments in more gentle environments. The sensors might also show cross sensitivity towards CO_2_ and other gasses that change during the experiment, and this might interfere with the calibration as well. The cross sensitivity should be of focus in future research.

## 4. Conclusions

The described O_2_/temperature sensors show major perspectives within post harvest research and determination of respiration rates, and the physical set-up of the sensors worked very well. The sensors were able to work from within climate chambers and could transmit signals to the receiver. However, due to fall-outs of some sensors extra replicates must be considered. The sensors had a higher uncertainty in measuring the oxygen content than the reference method used, this could be improved by further calibration. The drawbacks of the sensors are their lack of low-level oxygen sensitivity and lack of CO_2_ information. There is a need for further development of chips that will make it possible to improve the sensors on these two points. The major advantage and reason for using the sensors is the ability to measure temperature and oxygen levels concomitantly and continuously. This enables fine-tuned determination of the temperature dependency of respiration leading to a better time-resolution of respiration. In a following paper, the sensors are used to determine respiration rates in broccoli and wild rocket harvested in different seasons and with varietal differences.

## References

[1] Fonseca SC, Oliveira FAR, Brecht JK (2002). Modelling respiration rate of fresh fruits and vegetables for modified atmosphere packages: A review. J. Food Eng.

[2] Lakakul R, Beaudry RM, Hernandez RJ (1999). Modeling respiration of apple slices in modified-atmosphere packages. J. Food Sci.

[3] Jacxsens L, Devlieghere F, De Rudder T, Debevere J (2000). Designing equilibrium modified atmosphere packages for fresh-cut vegetables subjected to changes in temperature. Lebensm.-Wiss. Technol.

[4] Mahajan PV, Oliveira FAR, Montanez JC, Frias J (2007). Development of user-friendly software for design of modified atmosphere packaging for fresh and fresh-cut produce. Innov. Food Sci. Emerg.

[5] Iqbal T, Rodrigues FAS, Mahajan PV, Kery JP (2009). Mathematical modeling of the influence of temperature and gas composition on the respiration rate of shredded carrots. J. Food Eng.

[6] Conesa A, Verlinden BE, Artes-Hernandez F, Nicolai B, Artes F (2007). Respiration rates of fresh-cut bell peppers under supertamospheric and low oxygen with or without high carbon dioxide. Postharvest Biol. Technol.

[7] Gomes MH, Beaudry RM, Almeida DPF, Malcata FX (2010). Modelling respiration of packaged fresh-cut ‘Rocha’ pear as affected by oxygen concentration and temperature. J. Food Eng.

[8] Hertog MLAT, Boerrigter HAM, van den Boogaard GJPM, Tijskens LMM, van Schaik ACR (1999). Predicting keeping quality of strawberries (cv. ‘Elsanta’) packed under modified atmospheres: An integrated model approach. Postharvest Biol. Technol.

[9] Pretel MT, Souty M, Romojaro F (2000). Use of passive and active modified atmosphere packaging to prolong the postharvest life of three varieties of apricot (*Prunus armeniaca* L.). Eur. Food Res. Technol.

[10] Torrieri E, Perone N, Cavella S, Masi P (2010). Modelling the respiration rate of minimally processed broccoli (*Brassica rapa* var. *sylvestris*) for modified atmosphere package design. Int. J. Food Sci. Technol.

[11] Kim JG, Luo YG, Gross KC (2004). Effect of package film on the quality of fresh-cut salad savoy. Postharvest Biol. Technol.

[12] Escalona VH, Verlinden BE, Geysen S, Nicolai BM (2006). Changes in respiration of fresh-cut butterhead lettuce under controlled atmospheres using low and superatmospheric oxygen conditions with different carbon dioxide levels. Postharvest Biol. Technol.

[13] Manolopoulou H, Papadopoulou P (1998). A study of respiratory and physico-chemical changes of four kiwi fruit cultivars during cool-storage. Food Chem.

[14] Ke DY, Vangorsel H, Kader AA (1990). Physiological and quality responses of bartlett pears to reduced O_2_ and enhanced CO_2_ levels and storage-temperature. J. Am. Soc. Hortic. Sci.

[15] Torrieri E, Cavella S, Masi P (2009). Modelling the respiration rate of fresh-cut Annurca apples to develop modified atmosphere packaging. Int. J. Food Sci. Technol.

[16] Ruiz-Garcia L, Lunadei L, Barreiro P, Robla JI (2009). A review of wireless sensor technologies and applications in agriculture and food industry: State of the art and current trends. Sensors.

[17] Ruiz-Altisent M, Ruiz-Garzia L, Moreda GP, Lu R, Hernandez-Sanchez N, Correa EC, Diezma B, Nicolaï B, García-Ramos J (2010). Sensors for product characterization and quality of speciality crops—A review. Comput. Electron. Agric.

[18] Wang N, Zhang NQ, Wang MH (2006). Wireless sensors in agriculture and food industry—Recent development and future perspective. Comput. Electron. Agric.

[19] Damas M, Prados AM, Gomez F, Olivares G (2001). HidroBus (R) system: Fieldbus for integrated management of extensive areas of irrigated land. Microprocess. Microsyst.

[20] Nadimi ES, Sogaard HT, Bak T (2008). ZigBee-based wireless sensor networks for classifying the behaviour of a herd of animals using classification trees. Biosyst. Eng.

[21] Schumann AW, Miller WM, Zaman QU, Hostler KH, Buchanon S, Cugati S (2006). Variable rate granular fertilization of citrus groves: Spreader performance with single-tree prescription zones. Appl. Eng. Agric.

[22] Vivoni ER, Camilli R (2003). Real-time streaming of environmental field data. Comput. Geosci.

[23] Green O, Nadimi ES, Blanes-Vidal V, Jorgensen RN, Storm IMLD, Sorensen CG (2009). Monitoring and modeling temperature variations inside silage stacks using novel wireless sensor networks. Comput. Electron. Agric.

[24] Bochtis DD, Sørensen CG, Green O, Bartzanas T (2011). A diagnostic system for improving biomass quality based on a sensor network. Sensors.

[25] Cameron AC, Boylan-Pett WALT, Lee JULI (1989). Design of modified atmosphere packaging systems: Modeling oxygen concentrations within sealed packages of tomato fruits. J. Food Sci.

[26] Oms-Oliu G, Soliva-Fortuny R, Martin-Belloso O (2008). Physiological and microbiological changes in fresh-cut pears stored in high oxygen active packages compared with low oxygen active and passive modified atmosphere packaging. Postharvest Biol. Technol.

